# Mismatch-Driven CRISPR/Cas12a Biosensing of UV-Induced DNA Lesions for Environmental Solar Exposure Surveillance

**DOI:** 10.1021/acs.est.5c12461

**Published:** 2026-04-05

**Authors:** Yu-Wen Chen, David Septian Sumanto Marpaung, Ya-Yu Chen, Murali Mohana Rao Singuru, Min-Chieh Chuang

**Affiliations:** ‡ Department of Chemistry, 34890 Tunghai University, Taichung 407224, Taiwan; § International Ph.D. Program in Biomedical & Materials Science, Tunghai University, Taichung 407224, Taiwan; ∥ Department of Biosystems Engineering, Institut Teknologi Sumatera, Lampung Selatan 35365, Indonesia; ⊥ Sustainability Science and Management Program, Tunghai University, Taichung 407224, Taiwan

**Keywords:** ultraviolet radiation, CRISPR/Cas12a, dosimeter, photolesions, environmental monitoring

## Abstract

Monitoring environmentally relevant ultraviolet (UV) radiation is critical for understanding its biological impacts on ecosystems and human health. However, conventional UV dosimeters lack the molecular sensitivity to detect DNA-level damage that initiates such effects. Here, we present a CRISPR/Cas12a-based biosensing platform capable of quantifying solar UV exposure through the detection of UV-induced thymine dimers in DNA activators. This system harnesses mismatch-driven suppression of Cas12a activity, enabling a reduction in the fluorescence signal in response to UV-induced molecular lesions. The impact of thymine arrangement and the dimerization position of the activators on sensitivity were investigated. UV-induced diminution in Cas12a’s *trans*-cleavage efficiency (*k*_cat_/*K*_m_) was also characterized, revealing a 1.67-fold decrease as the UVB dose increased from 0 to 2 J/cm^2^. Under optimized conditions, the sensor achieved a detection limit of 0.029 J/cm^2^ for UVB and demonstrated high sensitivity to UVC. Field validation under natural sunlight showed a strong correlation with reference radiometric measurements, validating the biosensor’s accuracy and environmental relevance. The system’s sensitivity to low lesion densities, straightforward mechanism, and simple operation highlights its potential for environmental surveillance, human health risk assessment, and ecological monitoring in response to solar UV radiation.

## Graphical abstract

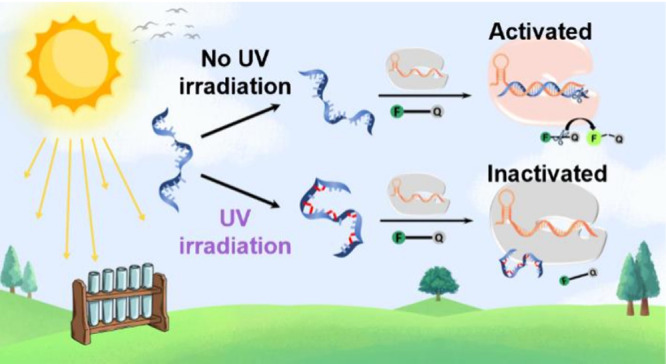

## 1 Introduction

The clustered regularly interspaced short palindromic repeats (CRISPR) and associated proteins (Cas), particularly the CRISPR/Cas9 and CRISPR/Cas12a systems, have enabled the successful development of genome-editing technologies and a variety of highly sensitive molecular diagnostic platforms.^1–3^ The canonical *trans*-cleavage activity of Cas12 requires precise *cis*-recognition, where crRNA forms perfectly matched base pairing with the target DNA, enabling specific *cis*-cleavage.^4,5^ This, in turn, activates the non-specific *trans*-cleavage of single-stranded DNA (ssDNA), a process known as DNA target-activated *trans*-DNase activity.^6^ The mismatch pairing between crRNA and the DNA activator regulates both the *cis*- and *trans*-activities of Cas12a. In double-stranded (dsDNA) activator, Cas12a binds tightly through two kinetically distinct steps: initial recognition of the protospacer-adjacent motif (PAM), followed by rate-limiting R-loop propagation that leads to cleavage of both DNA strands.^7^ Despite the functionally irreversible nature of this binding, Cas12a strongly discriminates against mismatches across the target sequence, with particularly high selectivity to variations within the PAM region.^8^ In contrast, Cas12a can be tolerantly activated by ssDNA through mismatched pairing with crRNA, exhibiting positional insensitivity across the entire activation region.^8,9^ However, this tolerance is influenced by the number of mismatches, and the reaction kinetics remain notably sensitive to single mismatches.^9,10^ These results highlight how mismatches induced by mispairing crRNA and the DNA activator can significantly influence the activity of CRISPR/Cas12a.

Thymine dimers are among the primary mutagenic photoproducts formed as a result of UV radiation exposure.^11^ These dimers can induce mismatched DNA pairing due to their low thermodynamic stability and weakened base-pairing affinity.^12,13^ Given the importance of taking preventive measures against the harmful effects of UV irradiation, researchers have leveraged this property to develop UV biological dosimeters that emulate the molecular damage seen in living systems, thereby providing biological insights that conventional physical or chemical dosimeters cannot offer.^14–16^

Per this feature, several biological dosimeters utilizing thymine dimer behavior have been developed, including those based on whole organisms,^17,18^ cells,^19,20^ and nucleic acids,^21,22^ all aiming to address the lack of biological relevance of purely physical- or chemical-based UV irradiation measurements.^23^ Among these, nucleic-acid-based systems are considered the most effective, as they eliminate variability associated with cellular metabolism, DNA repair, and organismal responses. Consequently, developing biodosimeters based on nucleic acid reactions holds significant practical utility and value.

In this study, we developed an UV monitoring strategy that integrates thymine dimer-containing DNA with the CRISPR/Cas12a system. This approach exploits the mismatch between crRNA and the dimerized DNA activator, wherein the formation of thymine dimers disrupts base pairing with the crRNA, thereby attenuating Cas12a activation. Given the intrinsically high catalytic activity of Cas enzymes and their sensitivity to mismatches between crRNA and target sequences, this mismatch-based mechanism enables the development of effective biosensing platforms.^10,24,25^ Furthermore, our design capitalizes on the robust *trans*-cleavage activity of Cas12a to establish a simple, amplification-free detection method, unlike other nucleic-acid-based biodosimeters that depend on PCR amplification or intricate, multidimensional DNA structures.^21,22,26^ To the best of our knowledge, this work represents a unique application of CRISPR/Cas12a for monitoring irradiation exposure doses. This mechanism offers a potent platform for quantifying solar UV exposure and broadens the potential applications of CRISPR technology in environmental sensing.

## 2 Materials and Methods

### 2.1 Reagents and Materials

The Lachnospiraceae bacterium Cas12a (Lba Cas12a) protein and NEBuffer r2.1 were obtained from New England Biolabs. CRISPR RNA (crRNA) and fluorophore-labeled reporter probes were synthesized by Integrated DNA Technologies (Coralville, IA, U.S.A.). DNA oligonucleotides were purchased from MDBio, Inc. (Taipei, Taiwan) and purified via PAGE. The sequences of all probes employed in this study are listed in Table S1. Cas12a was supplied by the manufacturer in liquid form and recommended for storage at −20 °C; however, it remains catalytically active for several months when stored in a lyophilized form at 4 °C. crRNA was provided in lyophilized form, stored at −20 °C, and rehydrated immediately prior to use. The DNA activator is chemically robust and can be stored in deionized water at 4 °C; nevertheless, lyophilization would further enhance its stability and extend its shelf life. Ultraviolet lamps, including UVA (F8T5BL), UVB (G8T5E), and UVC (GFT18DL) models from Sankyo Denki (Japan) as well as a LED light source (WLS-22-A, Mightex) were used for irradiation experiments. Real-time fluorescence detection was carried out on a QuantStudio 5 Real-Time PCR System (Applied Biosystems, Waltham, MA, U.S.A.). Fluorescence spectra were collected using a Shimadzu RF-6000 spectrofluorometer, while absorbance measurements for CPD concentration determination were obtained with a SpectraMax M2e microplate reader (Molecular Devices, U.S.A.).

### 2.2 UV Irradiation and Cas12a Reaction

Activator DNA (15 μL) was dissolved in deionized water and irradiated with ultraviolet light under defined power and exposure time conditions at 27 °C, maintained by a dry block heater. To ensure stable emission, the UV lamp was pre-warmed for at least 1 h prior to each experiment, and the output intensity was verified using a calibrated reference radiometer (SRI-2000, OPTIMUM). The dose values presented throughout the text and figures may vary slightly, typically by two or more decimal places across experiments, but these differences are not statistically significant. Following irradiation, the DNA solution was kept under ambient temperature and light conditions for approximately 10 min and then combined at a 1:1 volume ratio with a master mix containing NEBuffer r2.1, crRNA, Cas12a, and the ssDNA fluorescent reporter, yielding a final reaction volume of 30 μL. The final concentrations were 0.5 nM activator DNA, 30 nM crRNA, 30 nM Cas12a, 50 nM reporter, and 1× NEBuffer r2.1. Reactions were incubated at 37 °C for 60 min and subsequently heat-inactivated at 65 °C for 10 min. Fluorescence spectra were recorded by using an excitation wavelength of 495 nm.

### 2.3 Melting Curves, Polyacrylamide Gel Electrophoresis (PAGE), and Competitive ELISA

Monopoly-T_21_ was routinely used as the activator in melting curve analysis and PAGE and ELISA experiments. In general, activators were reconstituted in deionized water and exposed to varying doses of UVB irradiation prior to the analyses. To confirm the formation of T–T dimers [cyclobutane pyrimidine dimers (CPDs)], an ELISA kit (EU3586, FineTest) was employed. Detailed protocols for these experimental procedures are provided in the Supporting Information.

### 2.4 Catalytic Kinetics and Michaelis–Menten Analysis

Using activated Cas12a (1 nM monopoly-T_21_) with varying UV doses (0, 1, and 2 J/cm^2^) and reporter concentrations of 62.5, 125, 250, 500, and 1000 nM, the *trans*-cleavage kinetics assay was performed in a final reaction volume of 30 μL. Each CRISPR/Cas12a reaction mixture contained 30 nM Cas12a, 30 nM crRNA, and 1× NEBuffer r2.1. All *trans*-cleavage reactions were carried out at 37 °C, and fluorescence intensity was recorded every 60 s using a QuantStudio 5 Real-Time PCR System (Figure S1).

Linear regression was applied to the fluorescence signals at the 240th minute from Figure S1A and B against the reporter concentrations to obtain two slopes, representing 0% *trans*-cleavage activity (*S*_uncleavage_) and 100% *trans*-cleavage activity (*S*_cleavage_), respectively. Subsequently, *S*_uncleavage_ and *S*_cleavage_ were used in eq 1 to calculate the concentration of the *trans*-cleaved reporter, which was then plotted over time, as shown in Figure S2

1 $$c_{\mathrm{cl}}\left(t\right)\left(\mathrm{nM}\right)=\frac{\mathrm{RFU}\left(t\right)-c_{0}S_{\mathrm{uncleavage}}}{S_{\mathrm{cleavage}}-S_{\mathrm{uncleavage}}}$$

where *c*_cl_ represents the dynamic concentration of the *trans*-cleaved reporter, *c*_0_ is the initial concentration of the reporter, and RFU is the dynamic fluorescence value.

The slope obtained in the first 10 min (Figure S2B–D) was used to determine the initial reaction velocity (*V*_0_) of Cas12a. *V*_0_ values were then fitted to the applied reporter concentrations using the Michaelis–Menten enzyme kinetics model (eq 2) in OriginPro 2023, and the results are presented

2 $$V_{0}=\frac{V_{\max}\left[S\right]}{\left(K_{m}+\left[S\right]\right)}$$

where [*S*] represents the concentration of the reporter, *V*_max_ is the maximum reaction rate, and *K*_m_ is the Michaelis constant.

### 2.5 Environmental UV Monitoring

The oligonucleotide monopoly-T_21_ (0.5 nM, 15 μL) was first reconstituted in deionized water and transferred to a circular quartz cuvette covered with a quartz lid. The sample was then exposed to ambient sunlight. To prevent temperature-induced DNA degradation or water evaporation during sunlight exposure, the cuvette was placed over an ice–water bath. During the entire exposure duration, the sample and the reference radiometer (SRI-2000, OPTIMUM) were positioned side by side at the same horizontal level. We therefore assumed that the energy absorbed by the detection head was equivalent to that absorbed by the activator DNA. Furthermore, calibration was achieved by correlating the system’s fluorescence output with energy measured using the reference radiometer, indicating that the light field angular distribution effect was already incorporated into the energy quantification. The experimental setup is displayed in the Results and Discussion.

Solar exposure was conducted around noon to ensure consistent and strong irradiance. The reference radiometer recorded real-time irradiance spectra at 120 s intervals (Figure S3A). The total UV dose received by the sample was calculated by integrating the recorded irradiance values below 365 nm (Figure S3B) over the entire exposure duration and summing them to obtain the cumulative dose. Following solar exposure, the irradiated samples were analyzed according to the procedures described in section 2.2. To obtain the calibration curve for environmental solar monitoring, the signal decrease was plotted against the measured solar dose by using the reference radiometer.

## 3 Results and Discussion

### 3.1 UV-Induced Pyrimidine Dimer Activator for Activity Diminution of Cas12a

The working principle of the CRISPR/Cas12a-based UV dosimeter, driven by thymine dimer-induced mismatch and resulting activity attenuation, is illustrated in Figure 1A. The system utilizes a polyT activator composed of poly­(thymine) DNA sequences that hybridize with crRNA, thereby activating Cas12a. In the absence of UV irradiation, the activator maintains its native structure, allowing for efficient base pairing with the crRNA–Cas12a complex, which, in turn, triggers enzymatic activation. This activation induces Cas12a-mediated *trans*-cleavage of a single-stranded DNA (ssDNA) reporter, producing a detectable fluorescence signal. Upon UV exposure, adjacent thymine bases within the polyT sequence undergo dimerization, forming UV-specific photoproducts, such as pyrimidine–pyrimidone (6–4) photoproducts (6–4PPs) and cyclobutane pyrimidine dimers (CPDs).^11,12^ These lesions disrupt the complementary pairing between the polyT activator and crRNA, thereby inhibiting Cas12a activation. As a result, *trans*-cleavage activity is significantly reduced, leading to decreased ssDNA reporter cleavage and a corresponding decrease in the fluorescence intensity. This reduction in fluorescence, caused by Cas12a activity loss due to UV-induced DNA damage, serves as a quantitative indicator of the UV dose. Moreover, this response reflects biologically relevant DNA lesions, offering insight into UV exposure with direct implications for human health.

**Figure 1:**
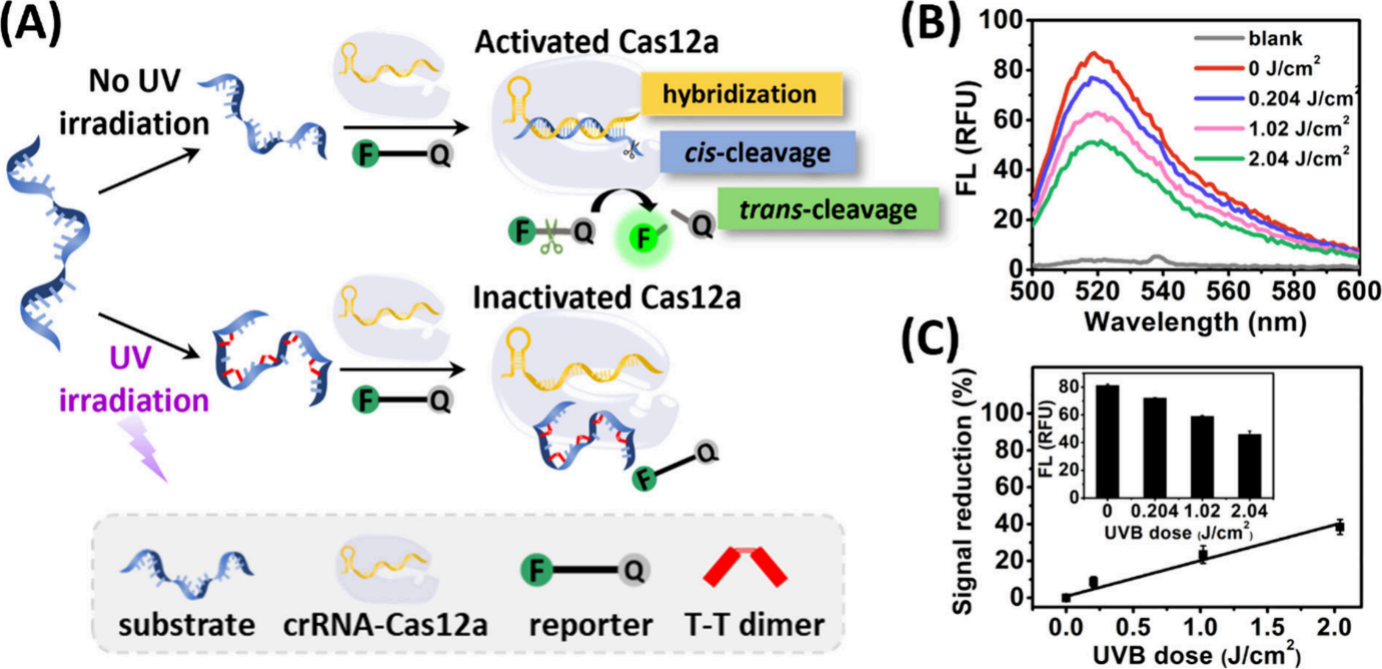
(A) Schematic illustration of the UV detection mechanism based on mismatch-driven CRISPR/Cas12a biosensing of UV-induced DNA lesions. (B) Fluorescence spectra of the system activated by monopoly-T_21_ at varying UVB exposure doses (0, 0.204, 1.02, and 2.04 J/cm^2^). (C) Corresponding fluorescence signal reduction for the data presented in panel B.

A DNA activator, monopoly-T_21_, consisting of 21 consecutive thymine residues flanked by 8-nucleotide fragments at both ends, was employed to assess the system’s response. As the UVB dosage increased, the fluorescence spectra of the *trans*-cleaved ssDNA reporter showed a progressive decline (Figure 1B), corresponding to a marked reduction in Cas12a activity. Fluorescence intensity was measured at 520 nm, and its decrease in response to increasing UVB exposure was normalized and expressed as a percentage of signal reduction calculated using eq 3.

3 $$\mathrm{signal reduction}\left(\%\right)=\frac{\left(F_{0}-F_{b}\right)-\left(F-F_{b}\right)}{\left(F_{0}-F_{b}\right)}\times 100\%$$

In this equation, *F*_0_ represents the fluorescence intensity without UV exposure, *F* denotes the fluorescence intensity at a given UVB dose, and *F*_b_ corresponds to the baseline fluorescence in the absence of the activator. The results demonstrated signal reductions of 23.5 and 38.4% at UVB doses of 1.02 and 2.04 J/cm^2^, respectively (Figure 1C). These findings reveal an approximately linear relationship between fluorescence signal reduction and UVB irradiation dose, underscoring the system’s potential as a reliable and sensitive UVB biodosimeter.

### 3.2 Thymine Dimer and Its Effect on the Binding Efficiency with crRNA

To validate the integrity of the detection system, we employed a CPD competitive enzyme immunoassay to confirm the formation of UVB-induced molecular lesions in the activator. As shown in Figure S4A, increasing CPD concentrations led to an exponential decrease in absorbance at 450 nm. This response, along with a calibrated standard curve (Figure S4B), was used to quantify the CPD levels in UVB-irradiated activators. Figure 2A demonstrates that the CPD concentration increased proportionally with UVB exposure of the monopoly-T_21_ activator, reaching 0.665 ng/mL at a UVB dose of 2.01 J/cm^2^. The corresponding lesion yield was 11.2% (Table S2), which is significantly higher than that of the 1TT activator, where a comparable yield required a UVB dose of 12.3 J/cm^2^ (Table S2). These results confirm the formation of CPD photoproducts in the poly­(thymine) DNA activator upon UVB irradiation, supporting the molecular basis of the CRISPR/Cas12a detection system.

**Figure 2:**
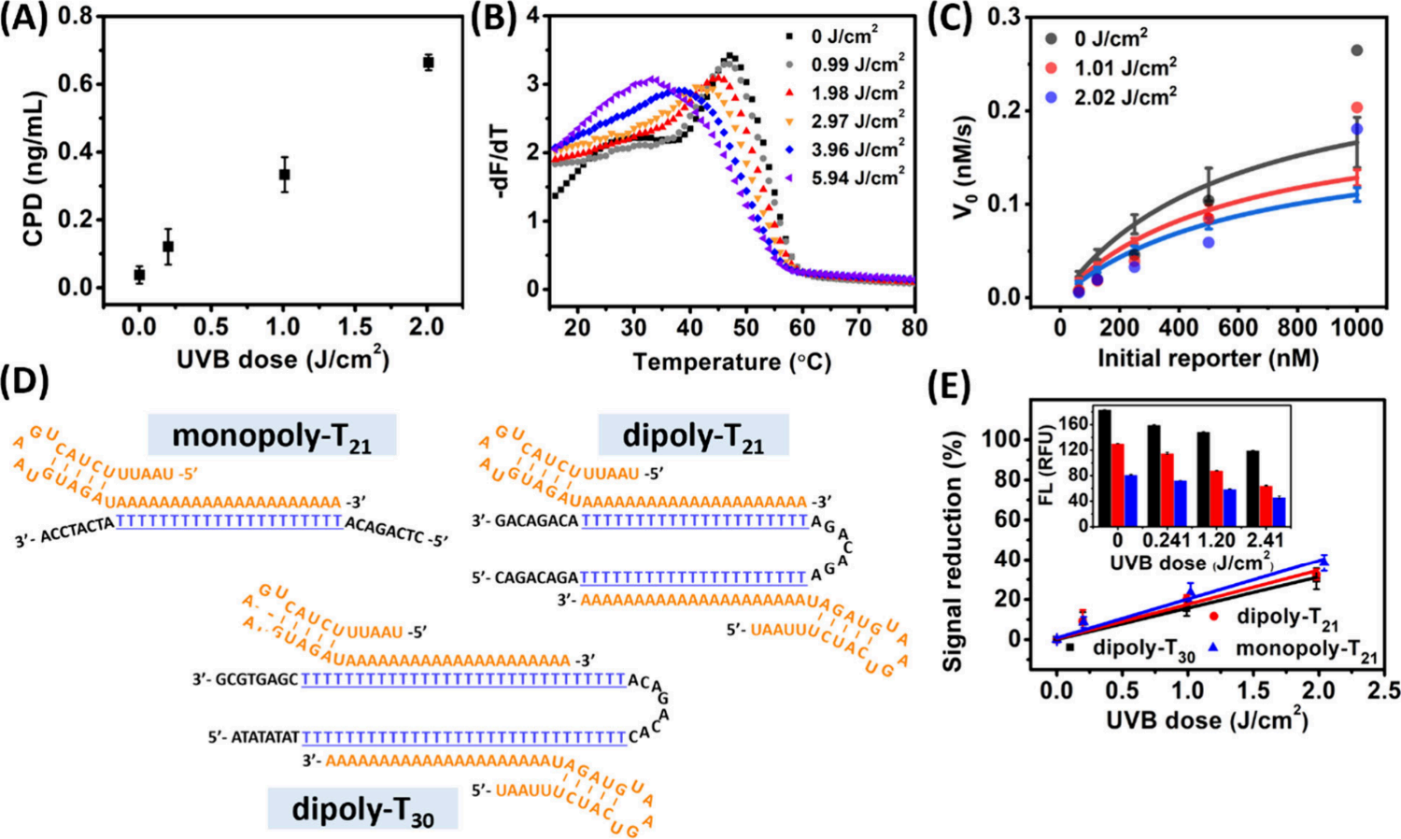
(A) CPD content in dimerized monopoly-T_21_ under various UVB exposure doses (0, 0.201, 1.01, and 2.01 J/cm^2^). (B) Melting curves of crRNA and monopoly-T_21_-exposed UVB at 0, 0.99, 1.98, 2.97, 3.96, and 5.94 J/cm^2^. (C) Michaelis–Menten plots of CRISPR/Cas12a-activated *trans*-cleavage activity using the monopoly-T_21_ activator under UVB doses of 0, 1.01, and 2.02 J/cm^2^. *V*_0_ denotes the initial catalytic rate. Reporter concentrations used were 62.5, 125, 250, 500, and 1000 nM. (D) Schematic illustration of monopoly-T_21_, dipoly-T_21_, and dipoly-T_30_ in complex with corresponding crRNA. (E) Signal reduction in *trans*-cleavage activity of monopoly-T_21_ (blue), dipoly-T_21_ (red), and dipoly-T_30_ (black) under varying UVB exposure doses (0, 0.241, 1.20, and 2.41 J/cm^2^).

While the formation of CPD photoproducts has been confirmed, the possibility that the observed changes in Cas12a activity may also arise from other UV-induced lesions, such as 6–4PPs, their Dewar isomers, or additional DNA damage, including oxidation and abasic site formation, cannot be excluded. Disentangling the individual contributions of these lesions would require enzymatic photolesion repair assays, which are technically more demanding and beyond the scope of this study, despite their potential to provide deeper insight into the mechanisms underlying the loss of *trans*-cleavage activity.

Elucidating the impact of thymine dimerization on crRNA binding efficiency can examine whether diminished Cas12a activity results from impaired hybridization between the UVB-irradiated activator and the crRNA–Cas12a complex. Melting curve analysis was performed to assess thermal dissociation behavior and base-pairing stability (Text S1). Prior to UVB exposure, the melting temperature (*T*_m_) of the crRNA–monopoly-T_21_ duplex was measured at 47 °C (Figure 2B). As UVB irradiation increased, *T*_m_ of the duplex progressively decreased, reaching 33 °C at a dose of 5.94 J/cm^2^. This indicates that the stability of crRNA–activator hybridization diminishes with increasing the formation of UV-induced photoproducts. To better mimic the actual hybridization environment within the CRISPR/Cas12a system, melting curves were also analyzed in the presence of Cas12a (Figure S5). The results revealed a *T*_m_ shift from 37 to 32 °C as UVB irradiation increased from 0 to 5.94 J/cm^2^. These findings confirm that thymine dimerization impairs the thermal stability of the crRNA–activator complex, ultimately reducing the catalytic efficiency of Cas12a.

### 3.3 Characterizations of Catalytic Activity

We further investigated the catalytic kinetics of Cas12a to understand how UVB exposure diminishes *trans*-cleavage activity through activator dimerization. Fluorescence signals (RFU) were converted to the corresponding concentrations of cleaved reporter molecules (Figures S1 and S2). As shown in Figure S2B–D, the concentration of the cleaved reporter increased over time but decreased with higher UVB doses, indicating a dose-dependent reduction in *trans*-cleavage activity. Notably, in the absence of UVB irradiation (Figure S2B), the cleaved reporter concentration was much higher than that observed after 2 J/cm^2^ UVB exposure (Figure S2D). Initial reaction velocities were plotted as a function of the reporter concentration (Figure 2C), revealing a clear decline in the reaction velocity with increasing UVB doses. The calculated turnover rates (*k*_cat_) of Cas12a were 0.053, 0.041, and 0.036 s^–1^ for activators exposed to 0, 1.01, and 2.02 J/cm^2^ UVB irradiation, respectively (Table 1). Furthermore, the *trans*-cleavage catalytic efficiency (*k*_cat_/*K*_m_) of Cas12a decreased significantly from 8.93 × 10^–5^ nM^–1^ s^–1^ (0 J/cm^2^) to 5.34 × 10^–5^ nM^–1^ s^–1^ (2 J/cm^2^), confirming UVB-induced impairment of enzymatic function.

**Table 1:** Catalytic Parameters of Cas12a with Activators Exposed to Varying UVB Dosage

UVB dose (J/cm^2^)	*V*_max_ (nM/s)	*k*_cat_ (s^–1^)	*k*_cat_/*K*_m_ (nM^–1^ s^–1^)
0	0.264 ± 0.052	0.053 ± 0.010	8.93 × 10^–5^ ± 9.15 × 10^–6^
1.01	0.203 ± 0.018	0.041 ± 0.004	6.94 × 10^–5^ ± 2.10 × 10^–6^
2.02	0.180 ± 0.007	0.036 ± 0.002	5.34 × 10^–5^ ± 9.64 × 10^–7^

The catalytic behavior of both *trans*- and *cis*-cleavage activities of Cas12a was also examined. As shown in the native PAGE results (Figure 3A), UVB exposure to monopoly-T_21_ significantly reduces *trans*-cleavage activity. A scrambled single-stranded DNA sequence (s-reporter, 45 nucleotides) was used as the reporter. Its characteristic band is clearly visible in lane 1. The band also remains unchanged in the presence of the crRNA–Cas12a complex alone (lane 2), indicating the absence of *trans*-cleavage in the absence of an activator. Upon the addition of monopoly-T_21_ to the crRNA–Cas12a complex, the s-reporter band disappears (lane 3), confirming successful activation of Cas12a and cleavage of the reporter. However, with increasing UVB doses (3.80, 7.60, 15.2, and 22.8 J/cm^2^), the s-reporter band progressively reappears (lanes 4–7), demonstrating a dose-dependent suppression of *trans*-cleavage activity.

**Figure 3:**
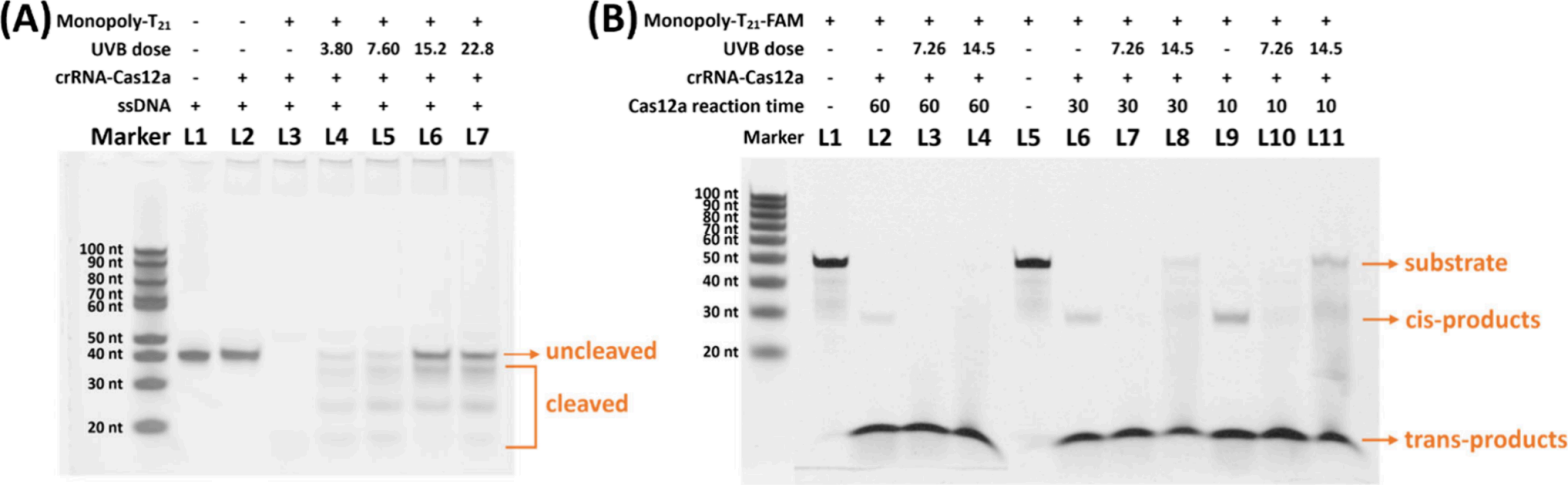
(A) Native-PAGE characterization of Cas12a *trans*-cleavage activity using monopoly-T_21_ at various UVB doses (3.80, 7.60, 15.2, and 22.8 J/cm^2^). Monopoly-T_21_, 5 nM; crRNA–Cas12a, 60 nM; and ssDNA (s-reporter), 1 μM. (B) Urea-PAGE characterization of Cas12a *cis*-cleavage activity using monopoly-T_21_-FAM at different UVB doses (7.26 and 14.5 J/cm^2^) and reaction times (10, 30, and 60 min). Monopoly-T_21_-FAM, 0.5 μM; crRNA–Cas12a, 1 μM.

To evaluate the *cis*-cleavage activity of Cas12a in response to varying UVB doses, a 3′-FAM-labeled monopoly-T_21_ activator (monopoly-T_21_-FAM) was used in urea-PAGE analysis (Figure 3B). The intact activator produces a distinct fluorescent band near 50 nucleotides (lanes 1 and 5) due to FAM labeling. In the presence of the crRNA–Cas12a complex, monopoly-T_21_-FAM undergoes *cis*-cleavage, resulting in the disappearance of the 50 nt band (lanes 2–4, 6–8, and 9–11), as the substrate is cleaved into two segments. One of these segments, containing the spacer region and the FAM label, is referred to as the *cis*-product, which initially binds to the crRNA–Cas12a complex. This binding is thought to protect the *cis*-product from being further cleaved at *trans*-cleavage sites within the Cas12a ternary complex.^27^ Upon denaturation with urea, the *cis*-product dissociates from the complex and appears as a band near 30 nt (Figure 3B).

Interestingly, the intensity of the *cis*-product band decreased with an extended reaction time, as seen by comparison of lane 2 (60 min), lane 6 (30 min), and lane 9 (10 min). This trend suggests that, with the extended reaction time, the *cis*-product is gradually displaced by fresh monopoly-T_21_-FAM during the catalytic cycle, leading to its release from crRNA and eventual digestion by *trans*-cleavage into smaller fragments (visible near the bottom of the gel). Importantly, the intensity of the *cis*-product band also correlated with the UVB dose. Higher UVB doses caused greater thymine dimer formation in monopoly-T_21_-FAM, impairing its hybridization with crRNA and thus reducing both *cis*- and *trans*-cleavage efficiencies. The diminished *trans*-cleavage activity allowed more intact *cis*-products to persist and be visualized on the PAGE gel. In addition, weaker binding between the dimerized activator and crRNA allowed the *cis*-product to dissociate more readily, resulting in stronger *cis*-product bands at higher UVB doses, as shown by comparisons between lanes 7 and 8 and between lanes 10 and 11.

### 3.4 Composition of PolyT Activators

Since thymine dimer formation in the activator affects CRISPR/Cas12a activity, we investigated how variations in the thymine segment length and arrangement within the DNA activator influence system performance. Specifically, three activator designs, monopoly-T_21_, dipoly-T_21_, and dipoly-T_30_, were evaluated (see Figure 2D), and their corresponding *trans*-cleavage fluorescence responses were systematically characterized. As shown in the inset of Figure 2E, increasing UVB doses led to progressive reductions in the fluorescence signals for all activators. When plotted as a function of UVB dose, the slopes of the calibration curves were 19.4, 17.1, and 15.7% J^–1^ cm^2^ for monopoly-T_21_, dipoly-T_21_, and dipoly-T_30_, respectively (Figure 2E), indicating that monopoly-T_21_ exhibits the highest sensitivity to UVB-induced suppression.

Monopoly-T_21_ consists of a single continuous poly­(thymine) segment, with the number of thymine residues exactly matching the number of adenines in the crRNA spacer region. This precise complementarity makes it highly susceptible to thymine–thymine (TT) dimer formation, whether clustered or dispersed, which introduces mismatches and disrupts crRNA binding, leading to a pronounced decrease in Cas12a activity. In contrast, dipoly-T_21_ and dipoly-T_30_ contain two separate thymine-rich regions, enabling multiple binding modes. As a result, even when TT dimers form in one segment, the crRNA–Cas12a complex may still recognize and bind another intact region, thereby partially preserving enzymatic activation. Furthermore, longer thymine segments (e.g., in dipoly-T_30_) offer additional potential binding sites. Even if photodamage occurs, unaffected 21 nt (or shorter^27^) stretches may still hybridize with the crRNA, reducing the system’s sensitivity to UV-induced inhibition. This compensatory binding behavior accounts for the lower sensitivity observed in multi-segment activators compared to single-segment monopoly-T_21_.

Given that the position of mismatches has been shown to significantly influence Cas12a activity,^8,9^ we investigated how the defined number and spatial arrangement of TT sites affect the sensitivity of this sensing mechanism. In contrast to the fully consecutive thymine sequence in monopoly-T_21_, an activator containing three dispersed TT motifs, referred to as 3TT (Figure S6A), exhibited notably lower susceptibility to UV-induced DNA damage, resulting in a reduced sensitivity of 4.40% J^–1^ cm^2^ (Figure S7). This reduced sensitivity is likely due to the limited capacity of 3TT to form only three TT dimer sites upon UVB exposure. Consequently, the degree of structural disruption in the crRNA–activator hybrid is minimal. The crRNA–Cas12a complex can still tolerate such imperfect hybridization, thereby limiting the overall signal reduction and system sensitivity.

### 3.5 Detection of a TT Dimer in Low Lesion Density

Understanding the ability of this system to sensitively recognize and detect even a single TT dimer in the activator is critical, as minimal DNA lesions caused by UV irradiation are often linked to pathological outcomes. To explore this, a pair of consecutive thymine bases was strategically positioned at three different locations within the DNA activator: the 5′ end (5′-1TT), the central region (1TT), and the 3′ end (1TT-3′), as illustrated in Figure S6B–D. These activators were subjected to UVB doses ranging from 0 to 12.2 J/cm^2^, and their *trans*-cleavage fluorescence signals progressively decreased with an increasing UVB exposure (Figure 4A). Despite containing only a single TT site, all three constructs exhibited a linear relationship between signal reduction and the UVB dose (Figure 4B). Quantitatively, the sensitivities were 4.41% J^–1^ cm^2^ for 1TT-3′, 4.30% J^–1^ cm^2^ for 1TT, and 3.64% J^–1^ cm^2^ for 5′-1TT. These results indicate that TT dimer formation near the 3′ end of the activator has the greatest inhibitory effect on Cas12a activity, followed by the middle and 5′ regions. This trend suggests that crRNA hybridization likely initiates from the 5′ end of the crRNA spacer and progresses toward the 3′ end. Therefore, structural disruptions near the 3′ end of the activator interfere more strongly with the hybridization process and, consequently, with Cas12a activation. This finding is consistent with previous studies on the positional effect of mismatches^8^ and highlights the sensitivity of the current method in detecting a single TT dimer, reinforcing its potential in detecting early-stage UV-induced DNA damage.

**Figure 4:**
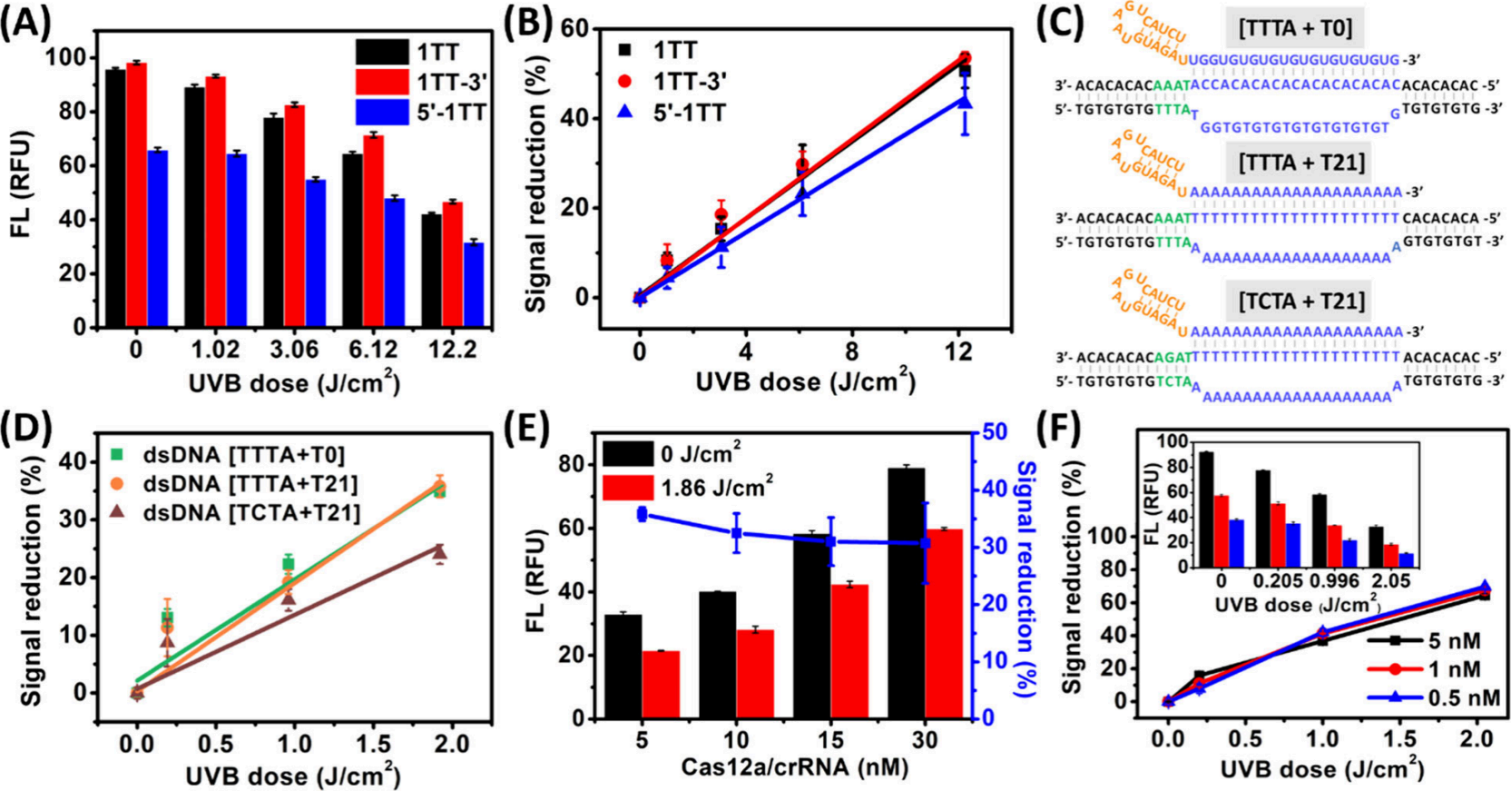
(A) *trans*-Cleavage fluorescence intensity of activators containing a single pair of consecutive thymine bases positioned at the 5′ end, center, or 3′ end of the sequence. (B) Corresponding fluorescence signal reduction derived from the data in panel A. (C) Schematic illustration of dsDNA activators with varied PAM and spacer region sequences. (D) Fluorescence signal reduction profiles for dsDNA activators with different PAM and spacer combinations. (E) Fluorescence intensity and corresponding signal reduction at varying concentrations of the crRNA–Cas12a complex. (F) Fluorescence signal reduction as a function of the monopoly-T_21_ activator concentration.

### 3.6 Double-Stranded Activators

Given that the crRNA–Cas12a complex exhibits catalytic activity toward both ssDNA and dsDNA activators, exploring its performance in UV irradiation sensing with dsDNA-based activators is particularly important for enhancing sensitivity. Building on insights gained from ssDNA activators, we designed dsDNA constructs in which TT dimer formation could occur within either the PAM site or the crRNA binding region, aiming to elucidate the structural elements most critical to suppressing Cas12a activity. Previous studies^28,29^ have shown that Cas12a prefers the TTTA sequence in the non-target strand (NTS) of the PAM region, which supports high *cis*- and *trans*-cleavage activity. Notably, TTTA contains consecutive thymine bases and is thus highly susceptible to UV-induced dimerization. Based on this, we engineered three dsDNA activators with distinct PAM and binding region sequences (Figure 4C), denoted as [A + B], where A represents the PAM sequence and B represents the crRNA binding region. A comparison of [TCTA + T21] and [TTTA + T21] revealed that [TCTA + T21] exhibited a lower sensitivity of 12.8% J^–1^ cm^2^ compared to 18.7% J^–1^ cm^2^ for [TTTA + T21] (Figure 4D). This reduction is likely due to the absence of a TT dimer formation potential at the PAM site. Moreover, the sensitivity of [TTTA + T0] was 17.6% J^–1^ cm^2^, comparable to that of [TTTA + T21], suggesting that TT dimer formation in the binding region exerts only a minimal effect when the PAM site contains a susceptible sequence, such as TTTA. These findings highlight the critical role of PAM structural integrity in modulating Cas12a activity. UV-induced mismatch within the PAM region results in the most significant reduction in enzymatic function, underscoring that PAM recognition is the essential initial step in Cas12a-mediated DNA targeting. Furthermore, the results confirm that Cas12a exhibits a low tolerance for structural alterations at the PAM site, reinforcing its sensitivity as a UV dosimeter.

### 3.7 Parameter Optimization in the CRISPR/Cas12a System

To further amplify the UVB-induced reduction in *trans*-cleavage fluorescence signals, we systematically optimized the concentrations of the Cas12a/crRNA complex and the monopoly-T_21_ activator. As shown in Figure 4E, when the Cas12a/crRNA molar ratio was fixed at 1:1 and varied from 5 to 30 nM in the presence of a UVB-irradiated activator (1.86 J/cm^2^), the overall fluorescence signal increased with higher Cas12a/crRNA concentrations. However, the signal reduction ratio, used to quantify UV-induced suppression, exhibited a slight decrease with an increasing Cas12a/crRNA concentration. This behavior is likely due to the limited availability of monopoly-T_21_ (1 nM): although more Cas12a/crRNA complexes are present, only a fixed number of activator molecules can hybridize and initiate cleavage. As a result, the sensitivity is not substantially improved. Based on these findings, 5 nM Cas12a/crRNA was selected for subsequent experiments. We evaluated the impact of the monopoly-T_21_ concentration on system performance. Increasing monopoly-T_21_ concentrations led to a proportional rise in the overall fluorescence signal (inset of Figure 4F). While the slopes of the calibration curves (signal reduction vs UVB dose) remained relatively similar across activator concentrations, differences became more pronounced at low UVB doses (0.205 J/cm^2^ in Figure 4F). At this level, 5 nM monopoly-T_21_ yielded the greatest signal reduction, followed by 1 and 0.5 nM. This behavior suggests that, at higher activator concentrations, more thymine residues are simultaneously susceptible to UVB-induced dimerization, resulting in greater TT dimer formation and more effective suppression of Cas12a activity. As UVB dosage continues to increase, the rate of TT dimer formation begins to plateau or saturate, diminishing the differences in signal reduction among varying activator concentrations. This saturation effect results in increasingly similar *trans*-cleavage suppression across conditions at high UVB doses.

### 3.8 Sensing Performance

Under optimized conditions, a strong linear relationship was observed between fluorescence signal reduction and UVB dose in the range of 0–0.186 J cm^–2^, with a correlation coefficient of *R* ^2^ = 0.998 (Figure S8). The limit of detection (LOD) was calculated to be 0.029 J cm^–2^ based on a signal 3 times the standard deviation (3σ) above the background signal. This LOD is superior to those reported in previous studies^13,18^ and comparable to other advanced biodosimeters.^26,30^ The cumulative uncertainty associated with achieving this LOD was estimated to be 2.421% (Text S5). Beyond UVB detection, the platform also demonstrated sensitivity to UVC and UVA irradiation. As shown in Figure 5A, the system exhibited even higher sensitivity to UVC, with two distinct linear regions producing sensitivities of 769 and 260% J^–1^ cm^2^ within the ranges of 0–0.0319 and 0.0319–0.0956 J/cm^2^, respectively (Figure S9). These values are significantly higher than the 135% J^–1^ cm^2^ sensitivity observed for UVB irradiation (Figure S8). The underlying mechanisms by which UVC alters sensitivity may involve additional types of DNA damage beyond CPD and 6–4PP formation. In contrast, exposure to UVA (0–20.0 J/cm^2^) resulted in a lower sensitivity of 2.20% J^–1^ cm^2^, reflecting the relatively milder impact of longer wavelength UVA on the DNA structure (Figure S10). These results collectively demonstrate that shorter wavelength UV irradiation causes greater DNA damage, highlighting the capability of this CRISPR/Cas12a-based biosensor to monitor UV irradiation based on the DNA damage potential.

**Figure 5:**
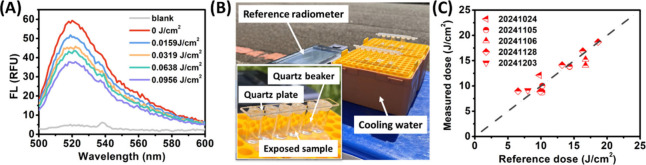
(A) Fluorescence spectra of the developed system in response to varying UVC doses (0–0.0956 J/cm^2^). (B) Schematic illustration of the experimental setup for detecting environmental sunlight. (C) Correlation between solar UV doses measured by the developed system and those obtained using a commercial dosimeter. Measurements were performed on 5 different days.

To determine the longest irradiation wavelength detectable by the current sensing system, we employed two light sources with narrower emission bandwidths and higher spectral precision: a 365 nm LED (emission peak centered at 364 nm; Figure S11) and a blue light source (emission at visible wavelength; Figure S11). Both were applied at varying irradiation doses to the system. As shown in Figure S12, exposure to 365 nm LED light caused a slight decrease in fluorescence with increasing dose (0–3.50 J/cm^2^), though the signal reduction showed limited linearity with respect to dose (Table S3). In contrast, the blue light source (emission peak near 465 nm with a bandwidth of 30 nm, with a dose range of 0–0.0839 J/cm^2^) produced no appreciable change in fluorescence intensity (Figure S13 and Table S4). These findings indicate that this CRISPR/Cas12a-based sensing system using monopoly-T_21_ as an activator is capable of detecting UV-induced DNA damage at wavelengths up to approximately 365 nm but not at longer wavelengths.

### 3.9 Environmental UV Monitoring

To assess the feasibility of the CRISPR/Cas12a-based sensing system for environmental UV monitoring, we applied the developed platform to detect sunlight-derived UV doses (Figure 5B). As shown in Figure S11, the spectral irradiance of natural sunlight begins at approximately 325 nm, extending into the visible range. We therefore used the portion of solar irradiation between 325 and 365 nm, as measured by a commercial UV dosimeter, as the reference standard for calibration of our system. A strong linear relationship (*R* ^2^ = 0.998) was observed between the fluorescence signal reduction and the solar UV dose below 365 nm (Figure S14), indicating excellent sensitivity and accuracy under real-world conditions.

Based on the established calibration curve, we further evaluated solar UV exposure on 5 separate days across October and December (Figure 5C). The temperature and humidity data for the testing days are summarized in Figure S15. During sunlight exposure experiments, environmental contaminants adhering to the quartz cuvette or its quartz plate lid (Figure 5B) may alter the transmission or reflection of UV light reaching the sample. To minimize such variability, operators should thoroughly clean the surfaces of the quartz components before each experiment. Notably, these contaminants do not directly interact with the DNA sample, as the quartz lid provides a physical barrier, preventing particulates from entering the quartz cuvette. In addition, to protect the sample from temperature-induced DNA degradation or evaporation during exposure, a quartz cuvette was placed atop an ice–water bath. This setup helped maintain a stable sample temperature, minimizing thermal variation during solar exposure and mitigating both intra- and interday fluctuations in ambient environmental conditions. The UV doses measured by the CRISPR/Cas12a system showed a strong correlation with the reference values obtained from the commercial dosimeter, with a regression slope of 0.991, demonstrating the system’s high reliability for environmental UV quantification. These results confirm that the CRISPR/Cas12a-based biosensor can accurately detect and quantify sunlight-induced UV radiation, effectively addressing the limitations of conventional DNA-based dosimeters.^31,32^ Collectively, these findings position the platform as a robust, sensitive, and field-deployable tool for environmental surveillance and public health assessment of UV exposure.

Toward this realistic route, several challenges remain to be addressed. Biochemical reagents used in this assay, including the DNA activator, crRNA, and Cas12a, could be lyophilized to enhance shelf life, enabling simplified packaging and transportation with limited cold chain requirements. Smartphone-based fluorescence readers offer a promising portable alternative to laboratory-based cuvette readers, especially when coupled with low-cost optical filters and LED excitation modules for field measurements. The reaction could be pre-loaded into microfluidic cartridges or paper-based analytical devices, enabling one-step rehydration and reaction initiation upon sample addition. However, the physicochemical conditions of DNA in the solid phase differ markedly from those in aqueous environments or living systems. In particular, the absence of water may reduce the efficiency of photochemical and radiolytic processes, as water molecules play a critical role in mediating these interactive reactions. Automation of signal quantification through a dedicated mobile app would allow real-time UV dose readouts, data logging, and geotagging for longitudinal or spatial exposure tracking. Ultimately, coupling these technical advancements with regulatory validation and field trials will be essential for transitioning this biosensing platform from a laboratory proof of concept to a practical tool for environmental monitoring, occupational safety, and public health applications.

## 4 Implications

Environmental ultraviolet radiation poses well-established risks to ecosystems and human health, yet existing dosimeters primarily measure physical irradiance without capturing the biologically meaningful molecular effects of sunlight exposure, such as biological relevance^33–35^ and molecular specificity.^17,19^ In contrast, the CRISPR/Cas12a-based biosensing platform developed in this study quantifies solar UV exposure through the direct detection of thymine dimers in DNA, offering sensitivity to low lesion densities without the need for enzymatic amplification or complex readout instrumentation. Unlike previous DNA-based biosensors^26,31,32^ that depend on PCR, multistep chemistry, or high-sensitivity lab equipment, our system demonstrates a simple, programmable, and amplification-free mechanism compatible with field-deployable fluorescence detection. The ability to correlate UV dose with DNA photodamage enables more precise environmental monitoring of solar radiation and its potential risks to biological systems.^33,36,37^ Field validation under natural sunlight showed strong agreement with reference radiometry, confirming its practicality for outdoor use. By bridging the gap between environmental exposure and biological consequence, this approach provides a transformative tool for assessing UV-related health and ecological risks, especially in vulnerable environments, such as high-UV-index regions or sensitive ecosystems. Its simplicity and sensitivity make it a promising candidate for incorporation into future portable UV dosimetry platforms, offering a new route for assessing solar UV exposure and its biological impacts, with direct implications for public health protection, climate-sensitive exposure assessment, and ecological monitoring in high-UV environments.

## Supplementary Material



## Notes

Additional details on oligos (Table S1), melting curves (Text S1), PAGE (Text S2), ELISA assay (Text S3), statistical analysis (Text S4), cumulative uncertainty of LOD (Text S5), dynamic fluorescence curves (Figure S1), enzymatic kinetic analysis (Figure S2), cumulative UV dose calculation (Figure S3), results of CPD analysis (Figure S4), lesion yields (Table S2), melting analysis containing Cas12a (Figure S5), schematic diagrams for 3TT, 1TT, 5′-1TT, and 1TT-3′ (Figure S6), sensitivity of the 3TT activator (Figure S7), LOD estimation (Figure S8), sensitivity of UVC (Figure S9), sensitivity of UVA (Figure S10), spectral profile of light sources (Figure S11), sensing results under irradiation of 365 nm LED (Figure S12 and Table S3), blue-light-irradiated results (Figure S13 and Table S4), calibration curves in response to natural sunlight (Figure S14), and environmental temperature and relative humidity (Figure S15) (PDF)
