# Supplementary material for: Mismatch-Driven CRISPR/Cas12a Biosensing of UV-Induced DNA Lesions for Environmental Solar Exposure Surveillance
Source: Environ Sci Technol. 2026 Apr 5;60(22):15930–9. doi: 10.1021/acs.est.5c12461 (PMC13262038; doi:10.1021/acs.est.5c12461)
Supplement: Supplementary file 1 [file es5c12461_si_001.pdf]

Supporting Information

Mismatch-Driven CRISPR/Cas12a Biosensing of UV-Induced DNA Lesions for Environmental Solar Exposure Surveillance

Yu-Wen Chen[1,#], David Septian Sumanto Marpaung[2,3,#], Ya-Yu Chen[1], Murali Mohana Rao Singuru[1], Min-Chieh Chuang[1,2,4]*

[1] Department of Chemistry, Tunghai University, Taichung 407224, Taiwan

[2] International Ph.D. Program in Biomedical & Materials Science, Tunghai University, Taichung 407224, Taiwan

[3] Department of Biosystems Engineering, Institut Teknologi Sumatera, Lampung Selatan 35365, Indonesia

[4] Sustainability Science and Management Program, Tunghai University, Taichung 407224, Taiwan

*To whom correspondence should be addressed.

[#] The authors contributed equally to this work.

MCC: Phone: 886-4-23590121 ext. 32218; FAX: 886-4-23590426; E-mail: mcchuang@thu.edu.tw

Submitted to ***Environmental Science & Technology***

Table S1. Oligos used in this study.

Oligos	Sequence (5'→3')
dipoly-T_{30}	ATA TAT AT**T TTT TTT TTT TTT TTT TTT TTT TTT TTT TT**C ACA GAC A**TT TTT TTT TTT TTT TTT TTT TTT TTT TTT T**CG AGT GCG
dipoly-T_{21}	CAG ACA GA**T TTT TTT TTT TTT TTT TTT TT**A GAC AGA **TTT TTT TTT TTT TTT TTT TTT** ACA GAC AG
monopoly-T_{21}	CTC AGA CA**T TTT TTT TTT TTT TTT TTT TT**A TCA TCC A
crRNA-polyA	AlTR1-UAA UUU CUA CUA AGU GUA GAU AAA AAA AAA AAA AAA AAA AAA-AlTR2
3TT	CAG ACA GAG GA**T T**AG GA**T T**AG CA**T T**AG GAG ACA GAC A
crRNA-3TT	AlTR1-UAA UUU CUA CUA AGU GUA GAU UCC UAA UGC UAA UCC UAA UCC-AlTR2
1TT	CAG ACA GAC AGA CAT ATA **TT**A CAG ACA GAC AGA CAG A
crRNA-1TT	AlTR1-UAA UUU CUA CUA AGU GUA GAU UCU GUC UGU AAU AUA UGU CUG-AlTR2
5'-1TT	CAG ACA GA**T T**AT ACA TAC AGA CAG ACA GAC AGA CAG A
crRNA-5'-1TT	AlTR1-UAA UUU CUA CUA AGU GUA GAU UCU GUC UGU CUG UAU GUA UAA-AlTR2
1TT-3'	CAG ACA GAG ACA GAC AGA CAG ATA TA**T T**AC AGA CAG A
crRNA-1TT-3'	AlTR1-UAA UUU CUA CUA AGU GUA GAU UAA UAU AUC UGU CUG UCU GUC-AlTR2
NTS-TTTA+T0	TGT GTG TG***T TT**A* TGG TGT GTG TGT GTG TGT GTG TGT GTG TG
TS-TTTA+T0	CAC ACA CAC ACA CAC ACA CAC ACA CAC CA*T AAA* CAC ACA CA
crRNA-TTTA+T0	AlTR1-UAA UUU CUA CUA AGU GUA GAU UGG UGU GUG UGU GUG UGU GUG-AlTR2
NTS-TTTA+T21	TGT GTG TG***T TT**A* AAA AAA AAA AAA AAA AAA AAA GTG TGT GT
TS-TTTA+T21	ACA CAC AC**T TTT TTT TTT TTT TTT TTT TT***T AAA* CAC ACA CA

NTS-TCTA+T21	TGT GTG TG*T CTA* AAA AAA AAA AAA AAA AAA AAA TGT GTG TG
TS-TCTA+T21	CAC ACA CA**T TTT TTT TTT TTT TTT TTT TT***T AGA* CAC ACA CA
reporter	6-FAM/TTA TT/IABkFQ
monopoly-T$_{21}$-FAM	ATC ATC CAA TCA TCC ACA CCA **TTT TTT TTT TTT TTT TTT TTT** ATC ATC CA-FAM
s-reporter (for PAGE)	TCC AGT GGT AAT CTA CTG GGA CGG AAC AGC TTT GAG GTG CGT GTT

FAM: fluorescein; IABkFQ: Iowa Black® fluorescence quencher; AlTR1: protecting group at 5’; AlTR2: protecting group at 3’.

Sequences involved in hybridization between activators and the crRNA are underlined. Consecutive thymine residues are indicated in **bold.** The PAM sites are indicated in *italics*.

Text S1. Melting Curves

The activator monopoly-T_{21} was reconstituted in deionized water and exposed to varying doses of UVB irradiation. The crRNA was reconstituted in a buffer solution containing 10 mM Tris and 0.1 mM EDTA (pH 7.5), and then pre-mixed with NEBuffer r2.1 and SYBR Green I (Thermo Fisher) to form a NEBuffer r2.1/crRNA/SYBR Green I working solution. A volume (5 µL) of this mixture was combined with 15 µL of UVB-treated monopoly-T_{21} solution, yielding a total sample volume of 20 µL. The final concentrations were 45 nM for both monopoly-T_{21} and crRNA, and 1× for NEBuffer r2.1 and SYBR Green I.

The sample solution was first heated to 95 °C and maintained for 3 min, then gradually cooled to 15 °C at a rate of 0.1 °C per second, followed by a 3 min hold at 15 °C. After cooling, the temperature was increased from 15 °C to 95 °C at a rate of 1 °C per minute, with fluorescence signals recorded at each 1 °C increment. The resulting fluorescence intensity (F) was plotted as a function of temperature (T) and analyzed by first derivative transformation, generating a dF/dT vs. T curve to determine melting transitions.

Text S2. Polyacrylamide Gel Electrophoresis (PAGE)

The reaction mixtures, including CRISPR/Cas12a reactions, were analyzed by gel electrophoresis. For native PAGE, a 12% polyacrylamide gel in 1× TBE buffer was prepared to assess Cas12a trans-cleavage activity. For urea PAGE, 7 M urea was incorporated into the gel mixture to analyze Cas12a cis-cleavage activity. Prior to loading, the urea PAGE was pre-run at 50 V for 30 min. Each 30 µL reaction mixture was combined with 6 µL of DNA loading dye, and 10 µL of this mixture was loaded per well. The Bio-25 bp™ DNA Ladder (Protech, Taiwan) was loaded in the first lane as a molecular size reference. Native PAGE was run at 80 V for 2 h, while urea PAGE was run at 100 V for 50 min using a Mini-PROTEAN electrophoresis system (Bio-Rad). Following electrophoresis, gels were stained with 1× SYBR Gold (prepared in 1× TBE buffer) for 20 min and imaged using a gel documentation system.

Text S3. Determination of Cyclobutane Pyrimidine Dimers (CPDs) Using Competitive ELISA

An ELISA kit (EU3586, FineTest) was employed to confirm the formation of T–T dimers (cyclobutane pyrimidine dimers, CPDs) in monopoly-T_{21} following UVB exposure. This assay utilizes a competitive ELISA detection format. The microtiter plate provided with the kit is pre-coated with CPDs. During the assay, CPDs present in the standards or samples compete with the immobilized CPD on the solid phase for binding to a biotinylated detection antibody specific to CPD. After incubation, unbound components are washed away, and HRP-conjugated streptavidin is added to each well and further incubated. Following this, TMB substrate solution is introduced to initiate a colorimetric enzymatic reaction. The reaction is then stopped by the addition of an acid solution, resulting in a color change that is measured spectrophotometrically at 450 nm. The CPD concentration in each

sample is determined by comparing its absorbance (OD_{450}) to a standard calibration curve. As this is a competitive assay, the concentration of CPD is inversely proportional to the OD_{450} value. Detailed procedures can be referred to the manufacturer's protocol.

Text S4. Statistical Analysis

All statistical analyses were conducted using OriginPro 2023. Data are presented as mean ± standard deviation (SD) with n = 3 for all replicates. The limit of detection (LOD) was estimated as a signal three times the standard deviation (3σ) above the background. This threshold signal was then converted to a UV dose using the linear regression model derived from fluorescence signal reduction as a function of UVB dose (Figure S8).

Text S5. Estimated cumulative uncertainty

$$CU = \sqrt{\sum (RSD_{Radiometer\ integration})^2 + (RSD_{Fluorescence\ quantification})^2 + (RSD_{Cas12a\ kinetics\ variation})^2} \quad \text{(S1)}$$

$$CU = \sqrt{\sum 0.424^2 + 1.56^2 + 1.803^2}$$

$$CU = 2.421\%$$

where,

CU	= Cumulative uncertainty of LOD (%)
$RSD_{Radiometer\ integration}$	= Relative standard deviation of radiometer integration (%)
$RSD_{Fluorescence\ quantification}$	= Relative standard deviation of fluorescence quantification (%)
$RSD_{Cas12a\ kinetics\ variation}$	= Relative standard deviation of Cas12a kinetic variation (%)

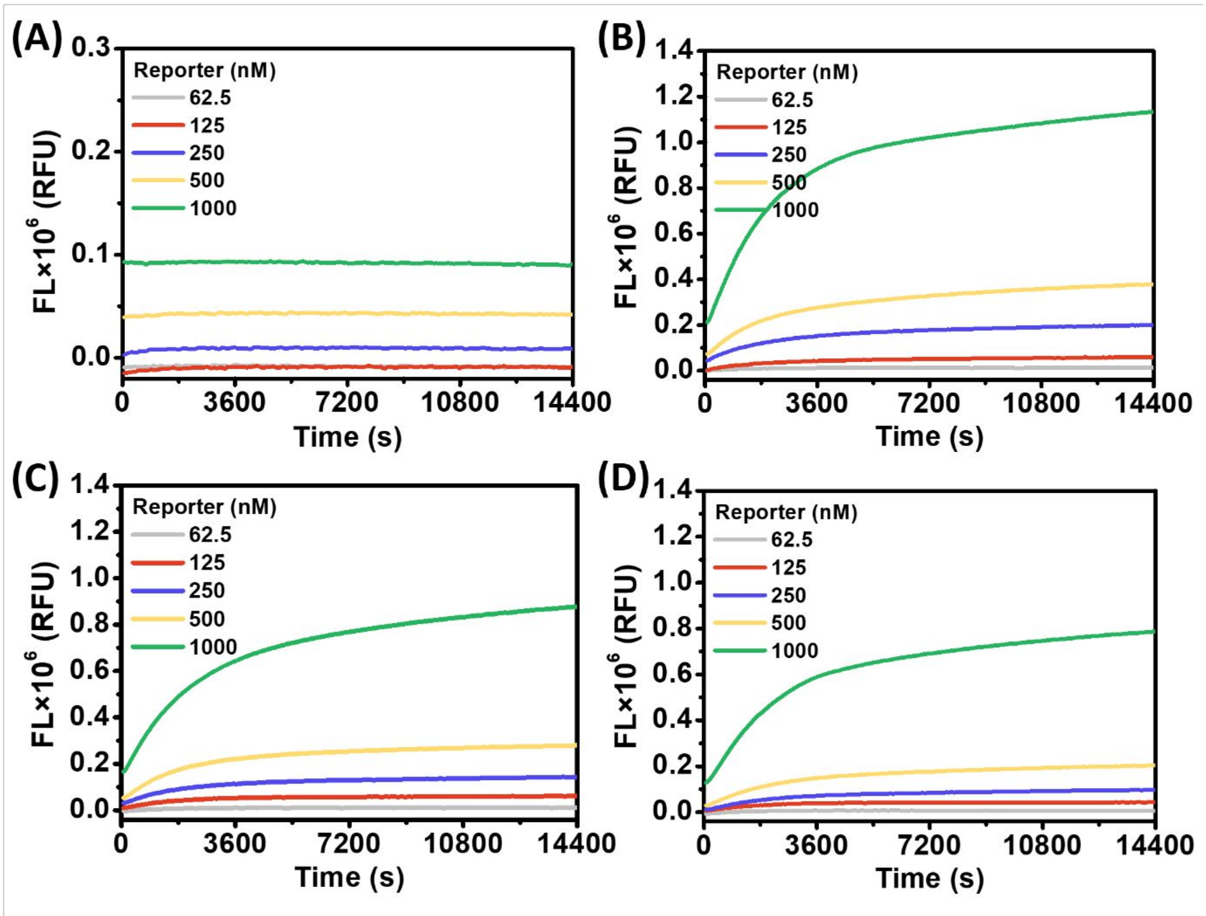

Figure S1. Fluorescence curves of monopolyT_{21}-activated CRISPR/Cas12a trans-cleavage reactions at reporter concentrations of 62.5, 125, 250, 500, and 1000 nM in the absence (A) and presence (B), (C), (D) of monopolyT_{21}, under 0 J/cm^2 (B), 1.01 J/cm^2 (C), and 2.02 J/cm^2 (D) UVB.

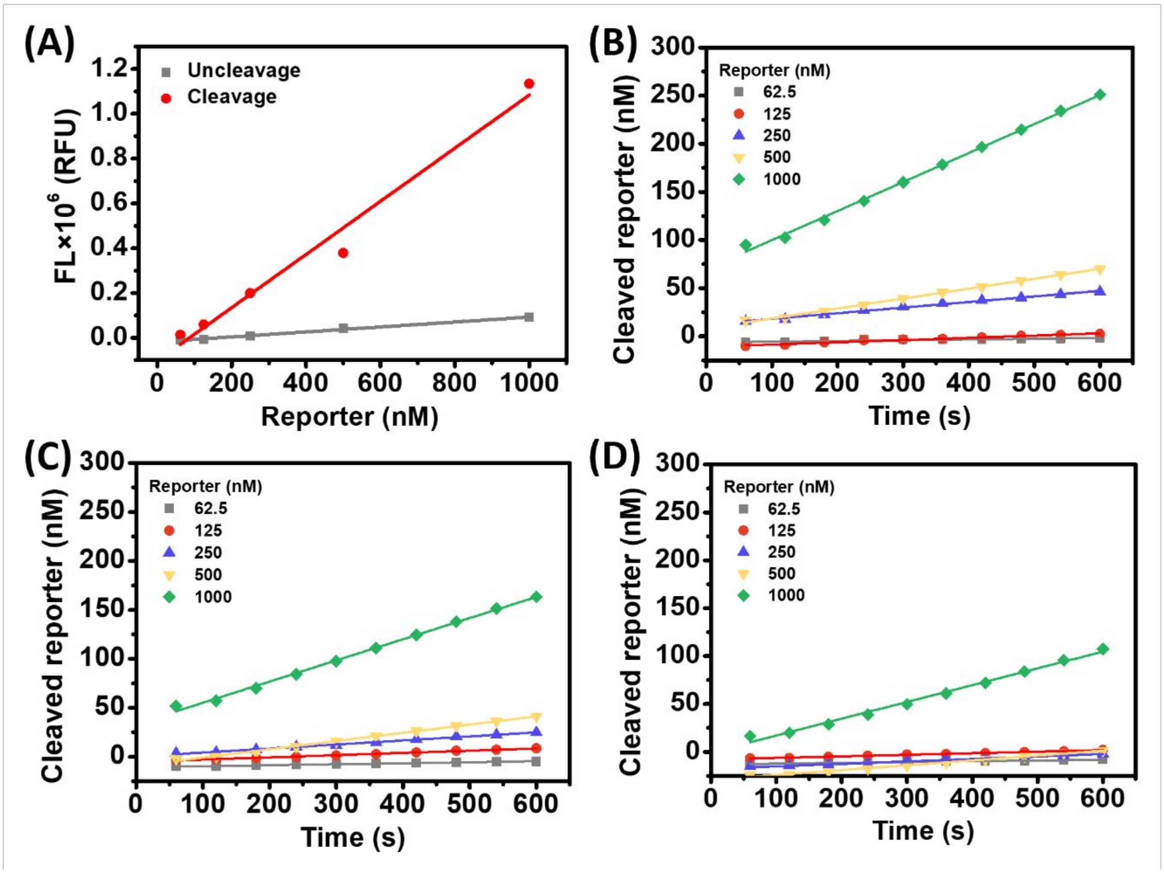

Figure S2. (A) Linear fitting of cleaved and uncleaved reporters. Cleaved reporter concentration of monopolyT_{21}-activated CRISPR/Cas12a trans-cleavage reactions at reporter concentrations of 62.5, 125, 250, 500, and 1000 nM under various UVB dose 0 J/cm^2 (B), 1.01 J/cm^2 (C), and 2.02 J/cm^2 (D). The first 10 minutes of cleaved reporter data were used for linear fitting to obtain the initial rate from the figure B–D.

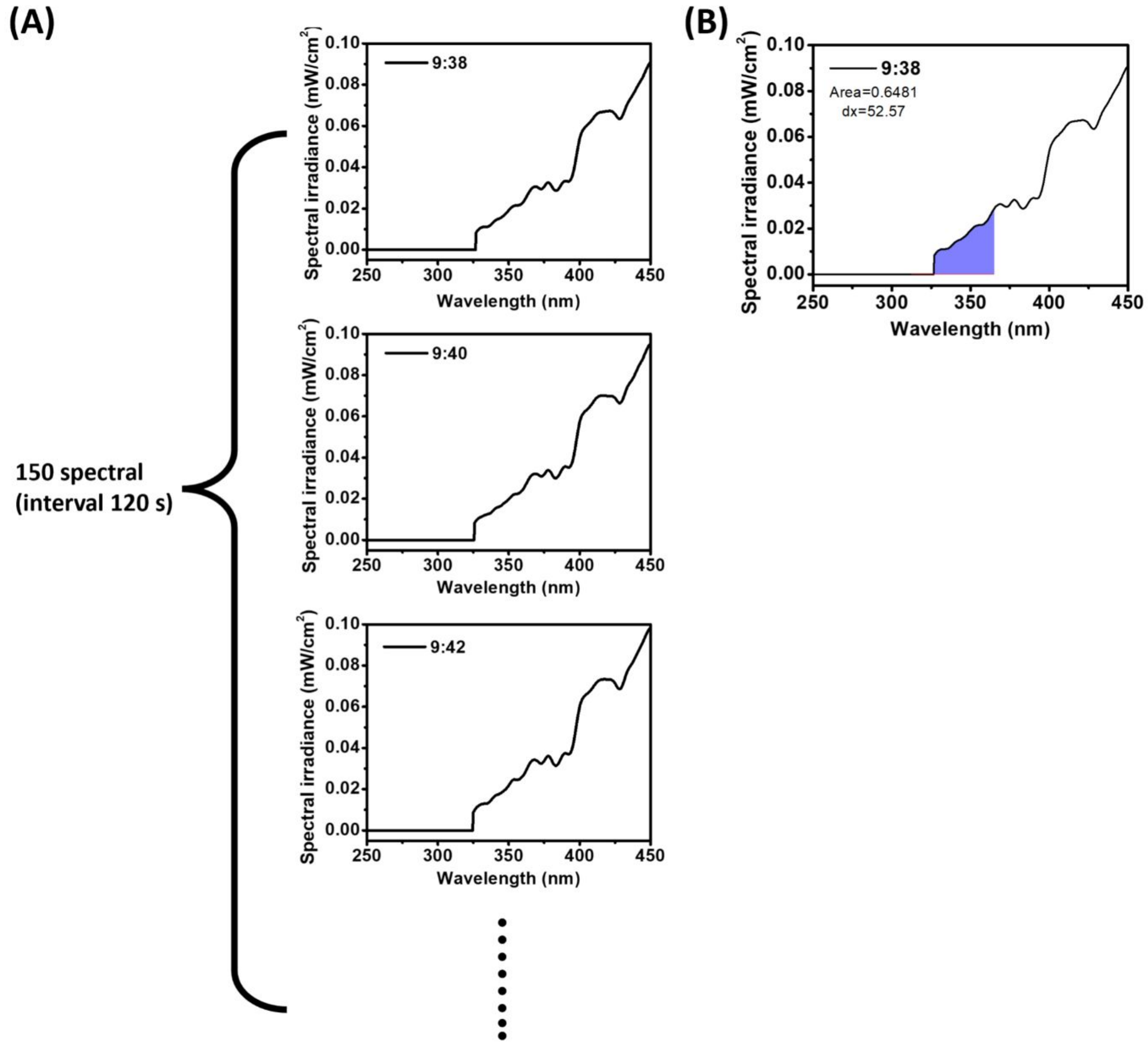

Figure S3. (A) Real-time irradiance spectra recorded at 120-second intervals using a reference radiometer (SRI-2000, OPTIMUM). (B) Cumulative UV dose calculated by integrating the recorded irradiance values below 365 nm (highlighted in purple) over the entire exposure period.

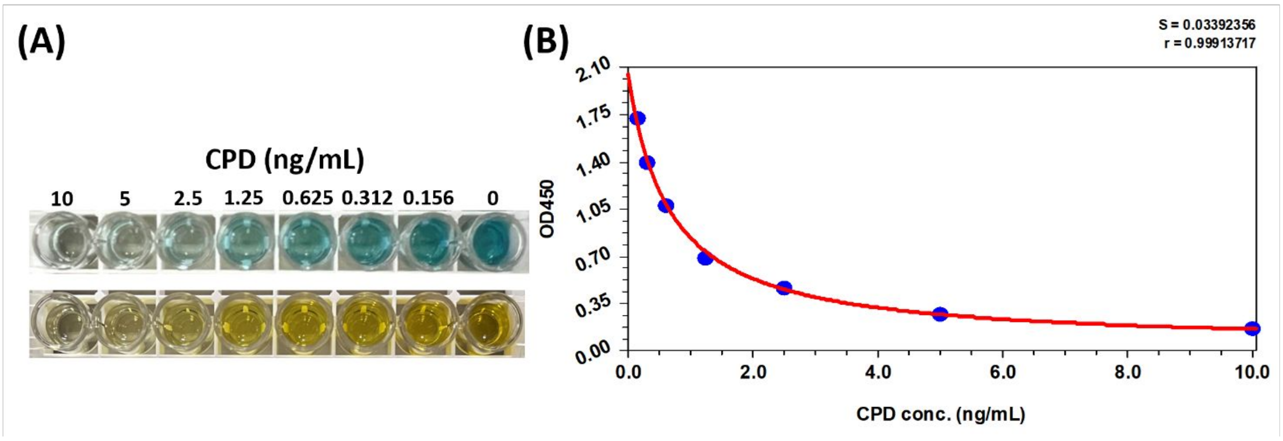

Figure S4. The calibration curve for standard CPD solutions. The CPD standard concentrations were 0, 0.156, 0.312, 0.625, 1.25, 2.5, 5, and 10 ng/mL.

Table S2. Lesion yields of activators monopoly-T_{21} and 1TT.

Activator type	UVB dose (J/cm^2)	CPD (ng/mL)	Lesion yield (%)
monopoly-T_{21}	0.201	0.0831	1.49
	1.01	0.296	5.29
	2.01	0.627	11.2
1TT	6.19	0.527	9.24
	12.3	0.661	11.6

$$Lesion\ yield\ (\%) = \frac{CPD \times 1000}{(Activator\ concentration\ \times MW)} \times 100\% \qquad \text{(S2)}$$

Where:

Lesion yield (%) : Percentage of CPD formation induced by UVB.

CPD (ng/mL) : Total cyclobutane pyrimidine dimers measured using the ELISA CPD kit in DNA photoproducts.

Activator concentration (nM) : Total concentration of DNA activator expressed in nanomolar units.

MW (g/mol) : Molecular weight of the activator expressed in grams per mole.

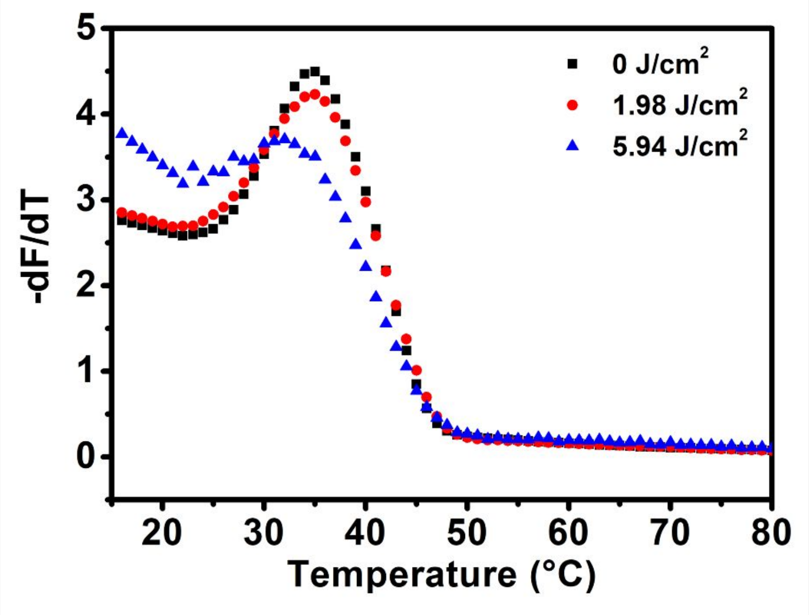

Figure S5. Melting curves of crRNA/Cas12a with monopoly-T_{21} which were exposed to UVB irradiation at 0, 1.98, and 5.94 J/cm^2.

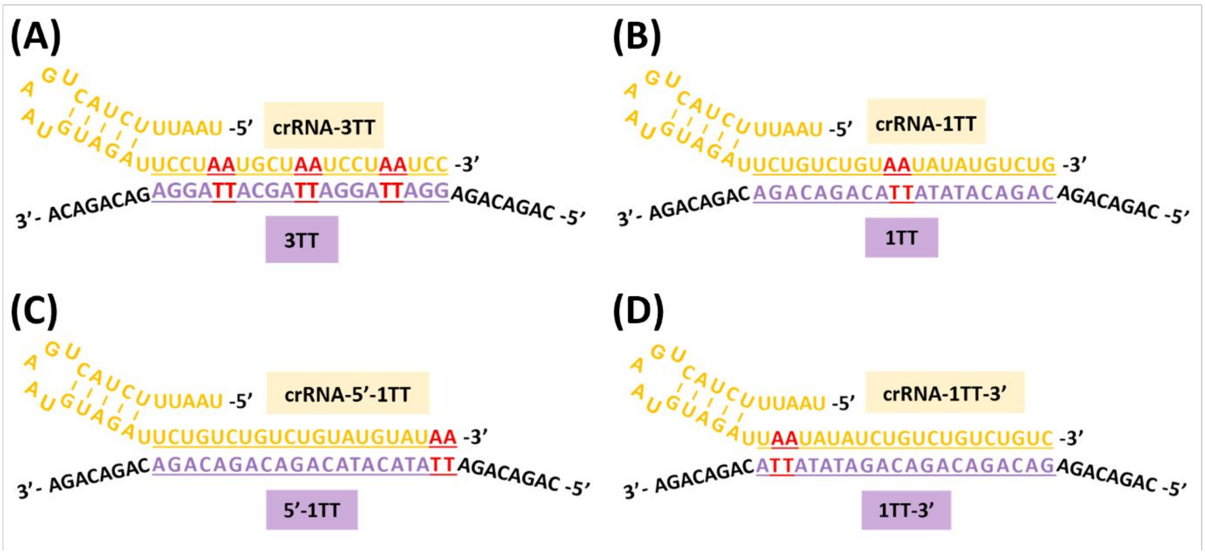

Figure S6. Schematic representation of different thymine–thymine positioning types: (A) 3TT; (B) 1TT; (C) 5′-1TT; (D) 1TT-3′

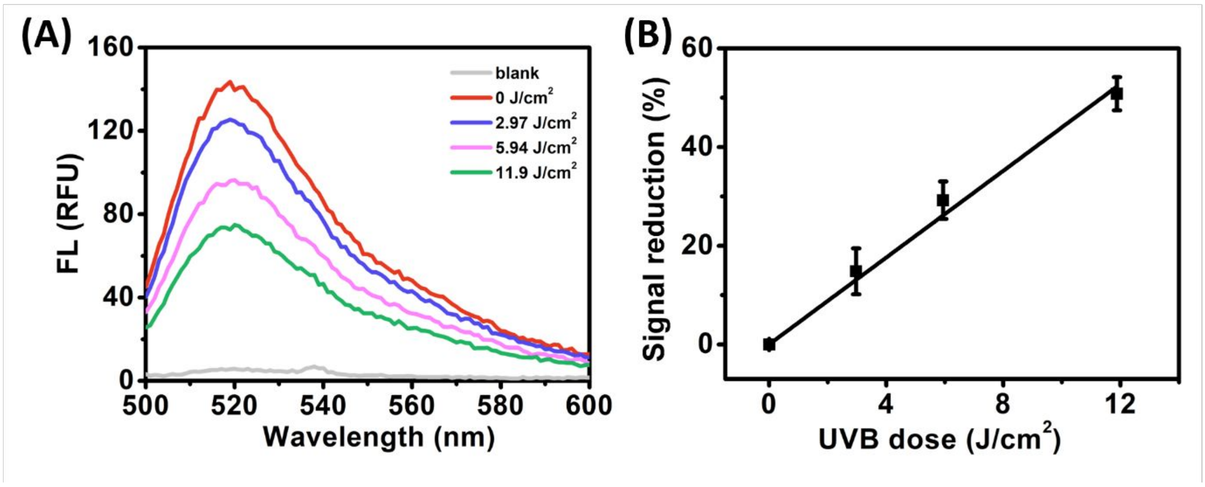

Figure S7. Sensitivity of UVB detection using the 3TT activator. (A) Fluorescence spectra at different UVB doses ranging from 0 to 11.9 J/cm^2. (B) Corresponding fluorescence signal reduction for the data presented in (A).

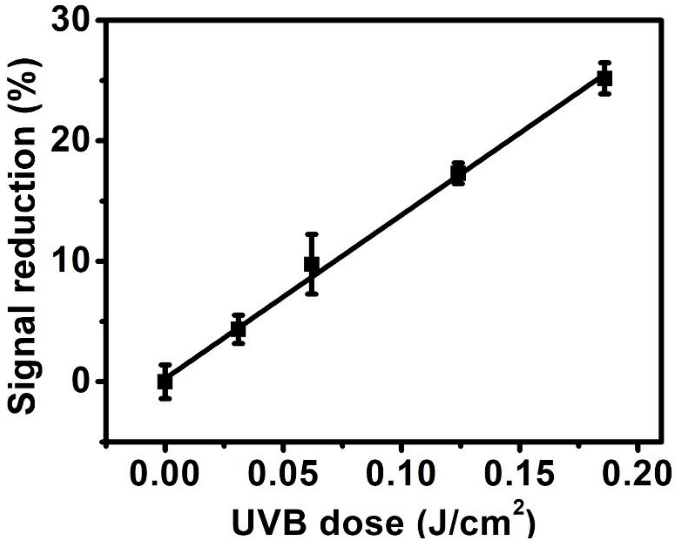

Figure S8. Fluorescence signal reduction at varying UVB doses ranging from 0 to 0.186 J/cm^2.

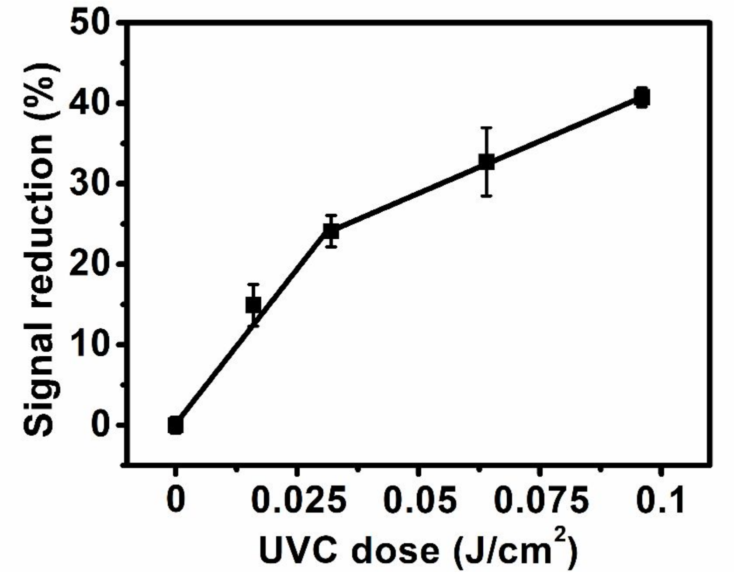

Figure S9. Fluorescence signal reduction at varying UVC doses ranging from 0–0.0319 J/cm^2 and 0.0319–0.0956 J/cm^2

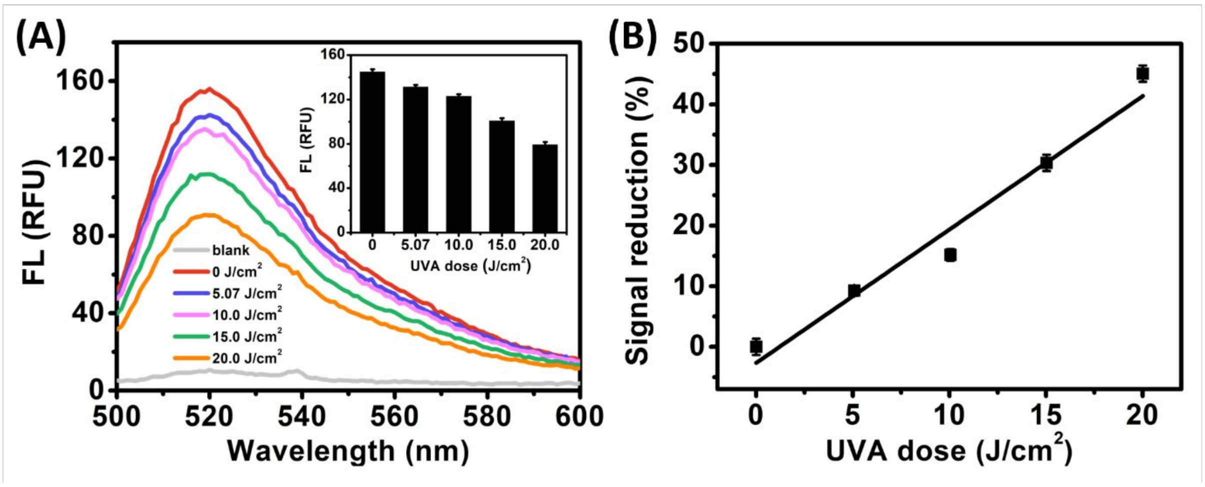

Figure S10. Sensitivity of UVA detection using the developed sensing system. (A) Fluorescence emission spectra at varying UVA doses ranging from 0 to 20.0 J/cm². (B) Corresponding fluorescence signal reduction as a function of UVA dose, based on the data presented in (A).

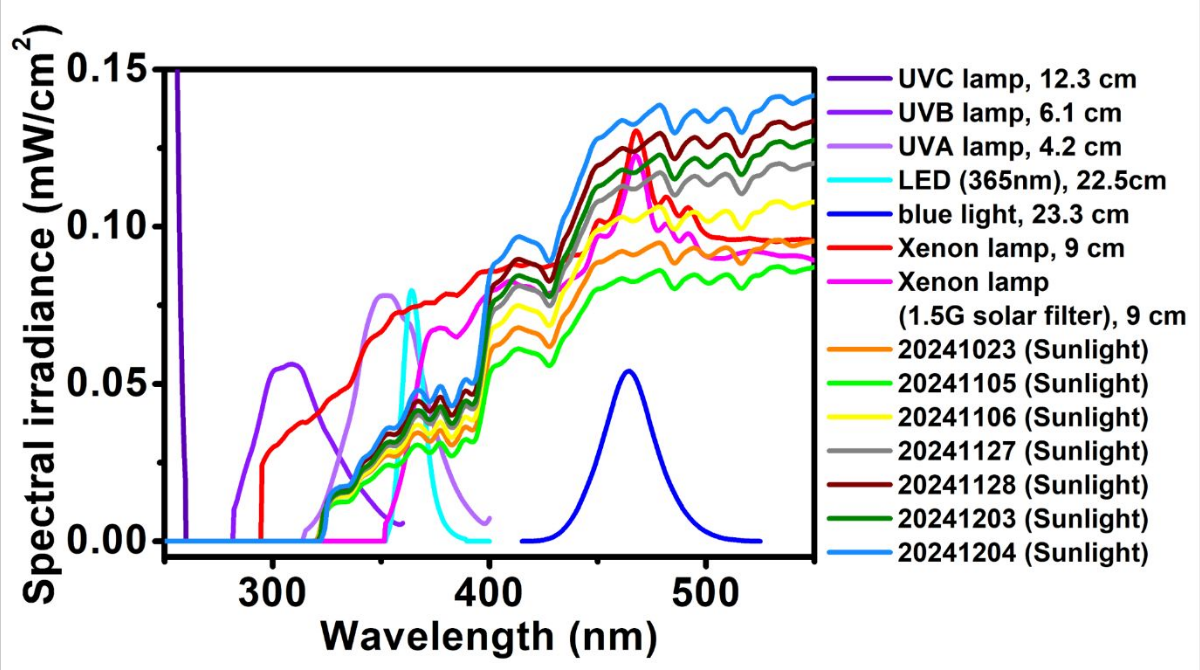

Figure S11. Spectral profile of light sources used and sunlight recorded using reference dosimeter (SRI-2000, OPTIMUM).

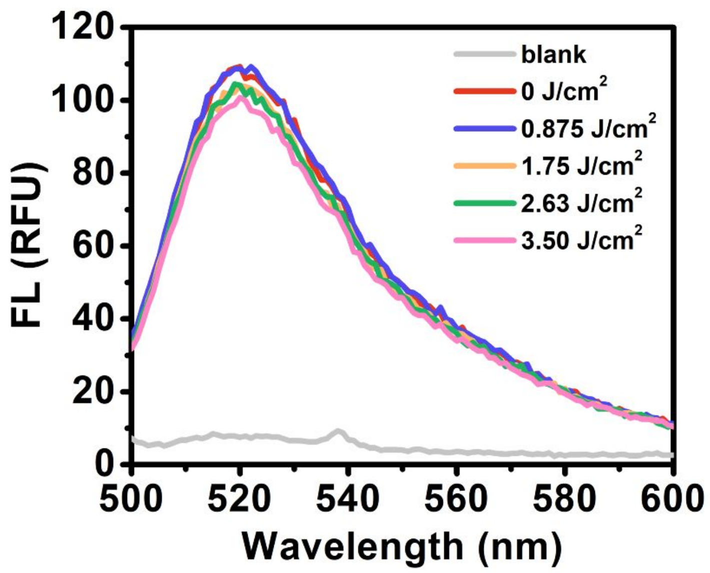

Figure S12. Fluorescence emission spectra of the developed CRISPR/Cas12a-based system at various LED light doses ranging from 0 to 3.50 J/cm^2.

Table S3. Fluorescence intensity values (n = 3) of the developed system as a function of LED light doses at 0, 0.875, 1.75, 2.63, and 3.50 J/cm^2.

LED (365 nm) (J/cm^2)	FL (RFU)			Avg.	SD
	Scan 1	Scan 2	Scan 3		
0	101.81	101.72	101.84	101.79	0.06
0.875	102.72	99.51	101.42	101.22	1.61
1.75	95.71	98.83	95.11	96.55	1.99
2.63	97.65	96.49	95.74	96.63	0.97
3.50	93.92	92.54	93.37	93.28	0.69

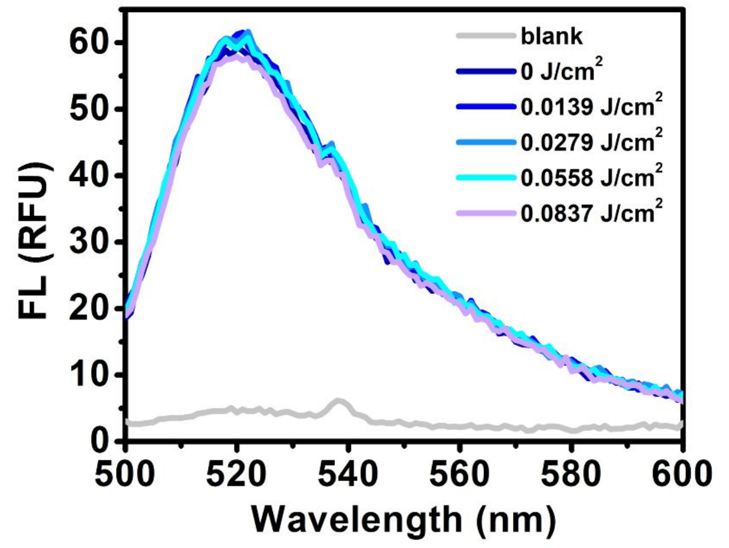

Figure S13. Fluorescence emission spectra of the developed CRISPR/Cas12a-based system under varying blue light doses ranging from 0 to 0.0837 J/cm^2.

Table S4. Fluorescence intensity values (n = 3) of the developed system as a function of blue light doses.

Blue light (J/cm^2)	FL (RFU) Scan 1	Scan 2	Scan 3	Avg.	SD
0	55.07	54.83	55.02	54.97	0.12
0.0139	56.45	57.06	57.07	56.86	0.35
0.0279	55.38	55.62	56.15	55.71	0.39
0.0558	54.80	54.45	55.00	54.75	0.28
0.0837	55.47	52.56	52.65	53.56	1.65

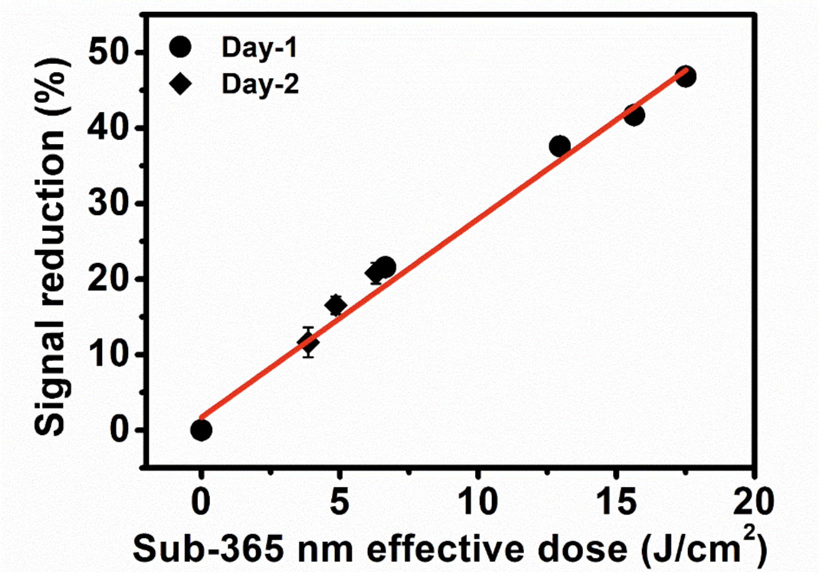

Figure S14. Fluorescence signal reduction of the developed CRISPR/Cas12a-based system in response to natural sunlight. Only irradiation with wavelength below 365 nm was considered for UV dose calculation.

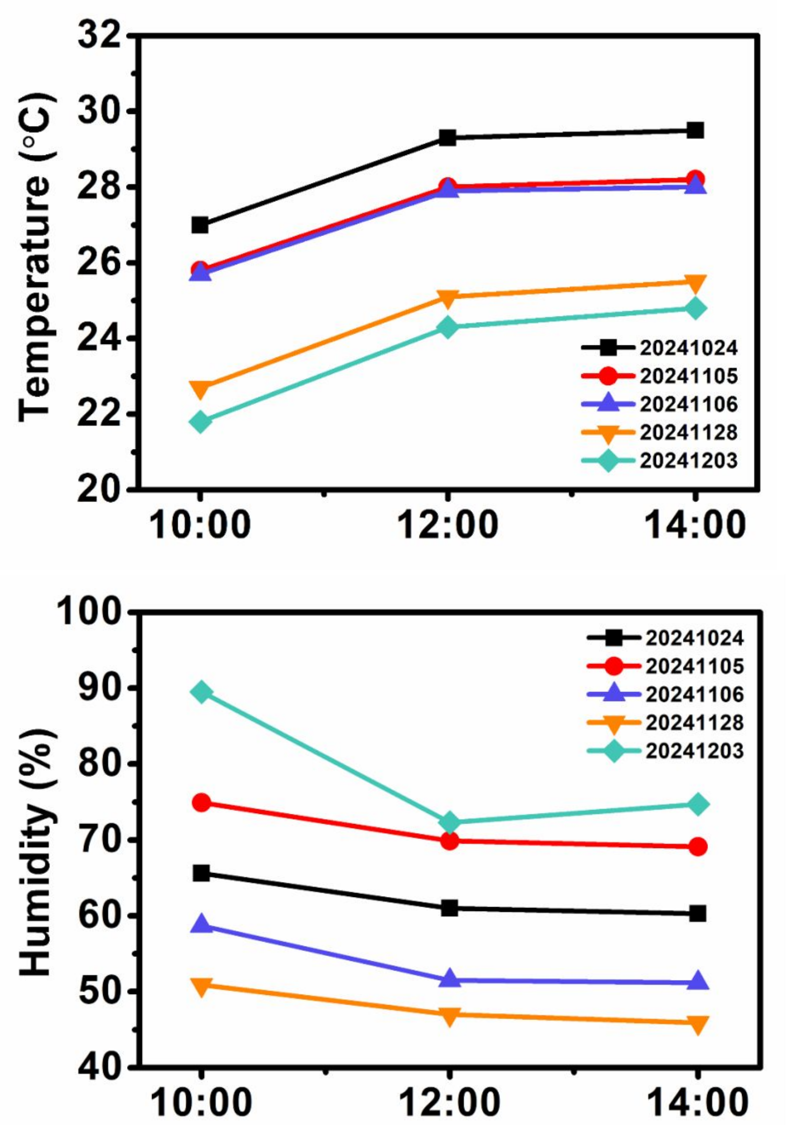

Figure S15. Environmental temperature and relative humidity recorded during the five days of solar UV dose measurements.
